# Employment and retirement impacts on health and wellbeing among a sample of rural Australians

**DOI:** 10.1186/s12889-021-10876-9

**Published:** 2021-05-10

**Authors:** Tonelle E. Handley, Terry J. Lewin, Peter Butterworth, Brian J. Kelly

**Affiliations:** 1 Centre for Rural and Remote Mental Health, University of Newcastle, University Drive, Callaghan, NSW 2308 Australia; 2 School of Medicine and Public Health, University of Newcastle, Callaghan, NSW Australia; 3 Centre for Brain and Mental Health Research, University of Newcastle, Callaghan, NSW Australia; 4 Centre for Research on Ageing, Health & Wellbeing, Australian National University, Canberra, ACT Australia

**Keywords:** Retirement, Employment, Ageing, Mental health, Wellbeing, Rural

## Abstract

**Background:**

In Australia, it is projected that one in four individuals will be at the nominal retirement age of 65 or over by 2056; this effect is expected to be especially pronounced in rural areas. Previous findings on the effects of retirement on wellbeing have been mixed. The present study explores the effects of employment and retirement on health and wellbeing among a sample of rural Australians.

**Methods:**

Australian Rural Mental Health Study participants who were aged 45 or over (*N* = 2013) were included in a series of analyses to compare the health and wellbeing of individuals with differing employment and retirement circumstances. Self-reported outcome variables included perceived physical health and everyday functioning, financial wellbeing, mental health, relationships, and satisfaction with life.

**Results:**

Across the outcomes, participants who were employed or retired generally reported better health and wellbeing than those not in the workforce. Retired participants rated more highly than employed participants on mental health, relationships, and satisfaction with life. There was also a short-term benefit for perceived financial status for retired participants compared to employed participants, but this effect diminished over time.

**Conclusions:**

While retirement is a significant life transition that may affect multiple facets of an individual’s life, the direction and magnitude of these effects vary depending on the retirement context, namely the pre-retirement and concurrent circumstances within which an individual is retiring. Personal perceptions of status changes may also contribute to an individual’s wellbeing more so than objective factors such as income. Policies that promote rural work/retirement opportunities and diversity and address rural disadvantage are needed.

**Supplementary Information:**

The online version contains supplementary material available at 10.1186/s12889-021-10876-9.

## Background

In Australia and internationally, the population structure is changing, with the proportion of individuals aged 65 and over projected to be the highest in recorded history in coming years [1]. This phenomenon will be among the most significant social transformations of the twenty-first century, with wide-ranging implications across many areas, including labour and financial markets, housing, and family structures and intergenerational ties [2]. Ensuring the health and wellbeing of older people will become increasingly important as this demographic group will account for a greater proportion of the population, while the resources to support them decrease [2]. Retirement represents a major life transition affecting older adults [3]. In Australia, it is projected that one in four individuals will be at the nominal retirement age of 65 or over by 2056 [4]. This effect is expected to be especially pronounced in regional and remote areas [5]; consequently, understanding the key associations between retirement and health and wellbeing in these locations is likely to be increasingly important, given the relative absence of existing research.

Current evidence regarding the effects of retirement on wellbeing lacks consensus, with a recent systematic review of twenty-two longitudinal studies on retirement and health reporting contradictory evidence across studies [6]. The authors concluded that further longitudinal research in this area is needed, particularly studies considering the contextual factors surrounding retirement, as this was noted to be lacking. Transition to retirement may also take several forms, including, for example, a single retirement episode (either voluntary or involuntary), multiple employment/retirement status changes over an extended period, or a shift from unemployment or other benefits to an age-based pension. These transition phases also need to be evaluated within the context of other key influences, such as chronic disease, recent adverse life events, and periods of unemployment, which have all been shown to impact on or be associated with physical, social, and emotional wellbeing [7–13], as have a range of other psychosocial factors [14, 15]. Early retirement may also be influenced by different factors to those associated with leaving the workforce at 65, and contribute to role uncertainty related psychological discomfort, at least in the medium term [16].

The relatively pervasive and reciprocal effects of unemployment on mental health are well known [17–19], but less is known about the health effects of other employment-related factors, such as the negative impact of job insecurity [20] or the positive impact of favourable psychosocial work conditions [21–23]. On the other hand, the observed positive effects associated with exiting from the workforce appear to be more marked among higher socio-economic groups [23].

In Australia, perhaps one of the most salient contextual factors is geographical location. Australia’s population is diverse, as residents inhabit a broad geographical area, and factors such as socio-demographic and socio-economic characteristics, community relationships, and the meaning and value of work often vary accordingly [24, 25]. Rural work, especially in certain industries such as farming, is often inter-generational and involves significant ties to the local community. The rural workforce is typically older than that in urban areas, with the average age of farmers being 53 years compared with 40 years for workers in other occupations [24]. Likewise, compared with major cities, rural areas have a greater proportion of residents aged over 65 [26]. The meaning of retirement itself among such diverse groups is worthy of exploration, together with changing societal attitudes and expectations. Moreover, cessation of an employment role can entail a significant impact on personal identity, as well as finances, property and housing, sense of purpose and belonging in the local community [25], and potentially initiates a major change in lifestyle. Compounding this, evidence suggests that rural residents are less likely to retire voluntarily, with forced retirement due to illness or injury being significantly more common than in urban areas [27]. Not only does this often necessitate a greater reliance on health and social support, it in turn is also associated with further negative outcomes, including poorer self-reported physical and mental health [28].

Considering the diverse contextual factors which may affect employment and retirement within rural areas, and the different domains of wellbeing in the post-retirement phase, the current analysis aims to build on previous research by exploring the effects of employment and retirement amongst a longitudinal cohort of rural Australians. Specifically, this research aims to: 1) examine health and wellbeing profiles among rural community-dwelling adults across various employment and retirement categories, with a focus on the determinants of perceived physical/functional and social/emotional outcomes; and 2) explore self-reported retirement plans/timeframes and the factors surrounding the decision to retire.

## Methods

### Participants

This analysis uses data from the Australian Rural Mental Health Study (ARMHS), which has been reported on previously [29, 30]. The ARMHS cohort was recruited based on the Accessibility/Remoteness Index of Australia Plus (ARIA+), selecting residents of inner regional, outer regional, remote and very remote New South Wales (NSW). The total ARMHS sample comprised household residents aged 18 years or older living in private dwellings. A total of 2639 participants enrolled in the study and completed baseline postal surveys between 2007 and 2008, with subsequent surveys completed at 12 months, 3 years and 5 years after baseline. Those who were lost to follow-up over the course of the study were younger and of a lower perceived financial position than those who completed all phases; no other baseline demographic or psychosocial characteristics were associated significantly with study attrition [10].

Participants were included in the present analyses if they were aged 45 years or older at baseline and had completed survey questions regarding their employment or retirement for at least one study phase.

### Socio-demographic, psychosocial and employment/retirement related measures

A broad range of socio-demographic, psychosocial, health and service use measures were utilised across the four ARMHS phases, including several standardised self-report instruments [29, 30]. However, only measures of direct relevance to the current analyses are detailed here, comprising either selected socio-demographic and psychosocial predictor variables, or possible confounders, potentially linked with work/retirement status, or global (single item) outcome measures assessing current perceived physical/functional and social/emotional health and wellbeing.

The ARIA+ [31], which was used to classify households in the current study, is based on the road distance to service towns, and reflects overall accessibility to goods, services and opportunities for social interaction. Five ARIA+ categories are typically used: Major cities (ARIA+ score ≤ 0.20) - relatively unrestricted accessibility; Inner regional (> 0.20 to 2.40) - some accessibility restrictions; Outer regional (> 2.40 to 5.92) - significantly restricted accessibility; Remote (> 5.92 to 10.53) - very restricted accessibility; and Very remote (> 10.53 to 15) - very little accessibility. Within the ARMHS data sets, increased remoteness has been shown to impact negatively on perceived personal and community drought-related stress [32]; however, relationships between hardship experiences and psychological distress also appear to be weaker in more remote locations [33].

Self-reported demographic information was obtained at each phase, including sex, date of birth, marital status, and whether they were living on a farm. A series of questions elicited employment/retirement status, occupation (including undertaking paid and unpaid work), reasons for not working, and related issues. An aggregate work/retirement status category was derived for each participant (see Table 1) based on responses to these questions *across the survey phases*: employed, not in workforce, or retired for all available phases; shifted from employed, or not in workforce, to retired (single transition); initially retired but re-entered workforce; or various categories involving multiple work/retirement changes across phases. Individuals initially coded as ‘not in workforce’ (e.g., unemployed, permanently unable to work, home duties) at a survey phase were automatically re-categorised as ‘retired’ once they reached 65 years of age.

**Table 1:** Participant characteristics for those aged 45 years or older at baseline (*N* = 2013)

Characteristic	Data source:		
	Baseline (***N*** = 2013) % (n)	All follow-ups (3366 surveys, ***N*** = 1469) % (n)	5-year follow-up (***N*** = 954) % (n)
**Work/retirement status**			
Employed (all available phases)	40.2 (810)	37.9 (1277)	37.9 (362)
Not in workforce (all available phases)	7.4 (148)	2.9 (98)	1.6 (15)
Retired (all available phases)	38.2 (769)	36.0 (1213)	33.8 (322)
Employed to retired	6.8 (136)	11.0 (370)	12.8 (122)
Not in workforce to retired	3.4 (68)	5.6 (187)	6.3 (60)
Initially retired - Re-entered workforce^a^	0.7 (15)	1.2 (40)	1.5 (14)
Initially employed - Multiple changes^a^	1.1 (22)	1.9 (64)	2.3 (22)
Not in workforce, then Multiple changes^a^	1.7 (35)	2.6 (89)	2.8 (27)
Initially retired – Multiple changes^a^	0.5 (10)	0.8 (28)	1.0 (10)
**Age** Mean (SD)	61.35 (10.00)	63.76 (9.67)	65.51 (9.26)
**Sex**			
Male	42.7 (860)	41.4 (1394)	40.3 (384)
Female	57.3 (1153)	58.6 (1972)	59.7 (570)
**Live on a farm**			
No	76.7 (1518)	79.5 (2642)	80.8 (761)
Yes	23.3 (461)	20.5 (682)	19.2 (181)
**Any chronic disease**			
No	46.9 (945)	38.1 (1284)	33.5 (320)
Yes	53.1 (1068)	61.9 (2082)	66.5 (634)
**Currently married**			
No	24.0 (480)	24.1 (806)	25.2 (238)
Yes	76.0 (1517)	75.9 (2541)	74.8 (706)
**Recent adverse life events** Mean (SD)	1.29 (1.29)	1.03 (1.14)	1.20 (1.24)
0–2	84.5 (1667)	89.6 (2997)	86.4 (822)
3–5	14.6 (288)	9.9 (331)	12.4 (118)
6–9	0.9 (18)	0.5 (18)	1.2 (11)
**ARIA** Mean (SD)	4.06 (3.14)	3.91 (3.07)	3.82 (2.98)
Inner regional	39.7 (800)	41.2 (1386)	41.3 (394)
Outer regional	36.1 (726)	36.9 (1241)	37.9 (362)
Remote	18.9 (380)	16.8 (566)	16.2 (155)
Very remote	5.3 (107)	5.1 (172)	4.5 (43)
**Occupation**			
Manager	17.0 (342)	18.5 (624)	19.1 (182)
Professional	16.9 (341)	20.0 (674)	21.4 (204)
Technician/trade worker	8.0 (162)	7.9 (267)	7.4 (71)
Community or personal service worker	7.0 (140)	7.7 (259)	8.3 (79)
Clerical or administrative worker	12.9 (260)	13.4 (451)	13.2 (126)
Sales worker	5.0 (100)	5.9 (197)	5.9 (56)
Machinery operator or driver	4.3 (86)	3.3 (111)	3.0 (29)
Labourer	6.8 (136)	6.1 (204)	5.3 (51)
Not in workforce	7.6 (153)	4.3 (144)	3.1 (30)
Mixed occupations	6.5 (130)	10.5 (353)	12.7 (121)
Missing	8.1 (163)	2.4 (82)	0.5 (5)

*Note*: ARIA, Accessibility/Remoteness Index of Australia Plus. A primary occupation category was derived for each participant based on all available surveys

^a^These sub-groups were excluded from the primary analyses due to small sample sizes and variable employment/retirement patterns

Current or previous occupation was also self-reported in a free-text question, with responses categorised according to Australian and New Zealand Standard Classification of Occupations (ANZSCO) categories. Where participants had reported more than one occupation across the four study phases, they were allocated to the occupation that they reported most consistently; participants who reported a different occupation at each phase were allocated to the ‘mixed occupation’ category.

Recent personal adverse life events were measured at each study phase using the 12-item List of Threatening Experiences [34]. Respondents were asked to indicate whether each event had occurred during the previous 12 months. In the current analyses, three recent life events items were excluded (relating to unemployment, downgrading at work, and major financial crises) because these factors were already assessed by other measures. The retained events covered: illness and deaths (among relatives and friends); arguments (within and outside of the household); serious accidents; court cases; and other adverse events.

The presence of chronic disease was determined by participants self-reporting any of the following: (‘Has a doctor ever told you that you have…’) heart attack or angina, other heart disease, high blood pressure, stroke, cancer, or diabetes. Previous ARMHS papers have examined associations between chronic disease status and wellbeing (among older rural residents) [11], quality of life [7], and lifetime affective and alcohol use disorder [35].

### Outcome measures

The outcome variables used here were derived from five ‘global rating of health’ measures. Global ratings of health were determined by a series of single-item questions developed for ARMHS. These questions began with the statement ‘during the past four weeks, how would you rate your…’, followed by five items: overall physical health; overall mental health; overall relationships; ability to perform everyday duties and tasks (identified subsequently as ‘everyday functioning’); and overall satisfaction with life. Responses were rated on 5-point scales ranging from ‘(1) poor’ to ‘(5) excellent’ or from ‘(1) not at all’ to ‘(5) extremely’. In some earlier ARMHS papers, standardised scores on these five measures were combined with other instruments to form an aggregate measure of current wellbeing [11, 29, 36], which tended to be higher among those aged over 65 years [11].

Perceived financial status was an additional outcome variable in the current analyses, and was measured by an item from the Household, Income and Labour Dynamics in Australia survey [37]: ‘Given your current needs and financial responsibilities, would you say that you and your family are: prosperous, very comfortable, reasonably comfortable, just getting along, poor, very poor?’ In the current analyses, this outcome measure was coded from ‘(1) poor or very poor’ to ‘(5) prosperous’.

Self-reported retirement status/plans and reasons for retirement were also assessed in the ARMHS 5-year follow-up survey. In response to the question ‘What were (or are likely to be) your main reasons for retiring?’, participants could choose from 13 alternatives (with no restrictions on the number of selections) and/or provide other reasons.

### Data analysis

Data were analysed using IBM SPSS (version 26; Armonk, NY, USA). Employment/retirement effects were explored in relation to six self-reported outcome variables, namely, perceived: physical health, everyday functioning, financial position, mental health, relationships, and satisfaction with life. For each of these outcomes, a separate generalised estimating equation (GEE) was run to explore the independent associations (reported as raw regression weights) with the following predictor variables: aggregate work/retirement status, study phase (and selected interactions by work/retirement status), sex, whether participants live on a farm, chronic illness (No/Yes), marital status (currently married: No/Yes), recent adverse life events (continuous score: 0 to 9), ARIA accessibility/remoteness (continuous score: 0 to 15), and current/previous occupation (11 categories).

As this analysis strategy differs somewhat from conventional analyses of longitudinal data (i.e., analyses examining differential changes in outcome variables over time), some additional explanation is warranted. By-and-large, our data set did not facilitate a prospective evaluation of employment transitions (during the actual study period), with the vast majority of participants (85.8%) in the same category at all study phases (i.e., employed, not in workforce, or retired); consequently, the reference category used in the GEE analyses for the dummy-coded work/retirement status predictor variables was the ‘Employed (all phases)’ group. However, there were sufficient numbers of participants who experienced a single transition during the study period (i.e., from employed, or not in the workforce, to retired) to enable some specific comparisons with the other work/retirement status groups. Aggregate differences between study phases and a targeted subset of interactions were also examined in the GEE analyses. For example, within-subject dummy-coded predictor variables assessing study phase differences were included, together with interaction predictors examining phase by work/retirement status effects; with the latter predictors only anticipated to reveal differential changes involving the transitioning groups (if they were present).

**Fig. 1:**
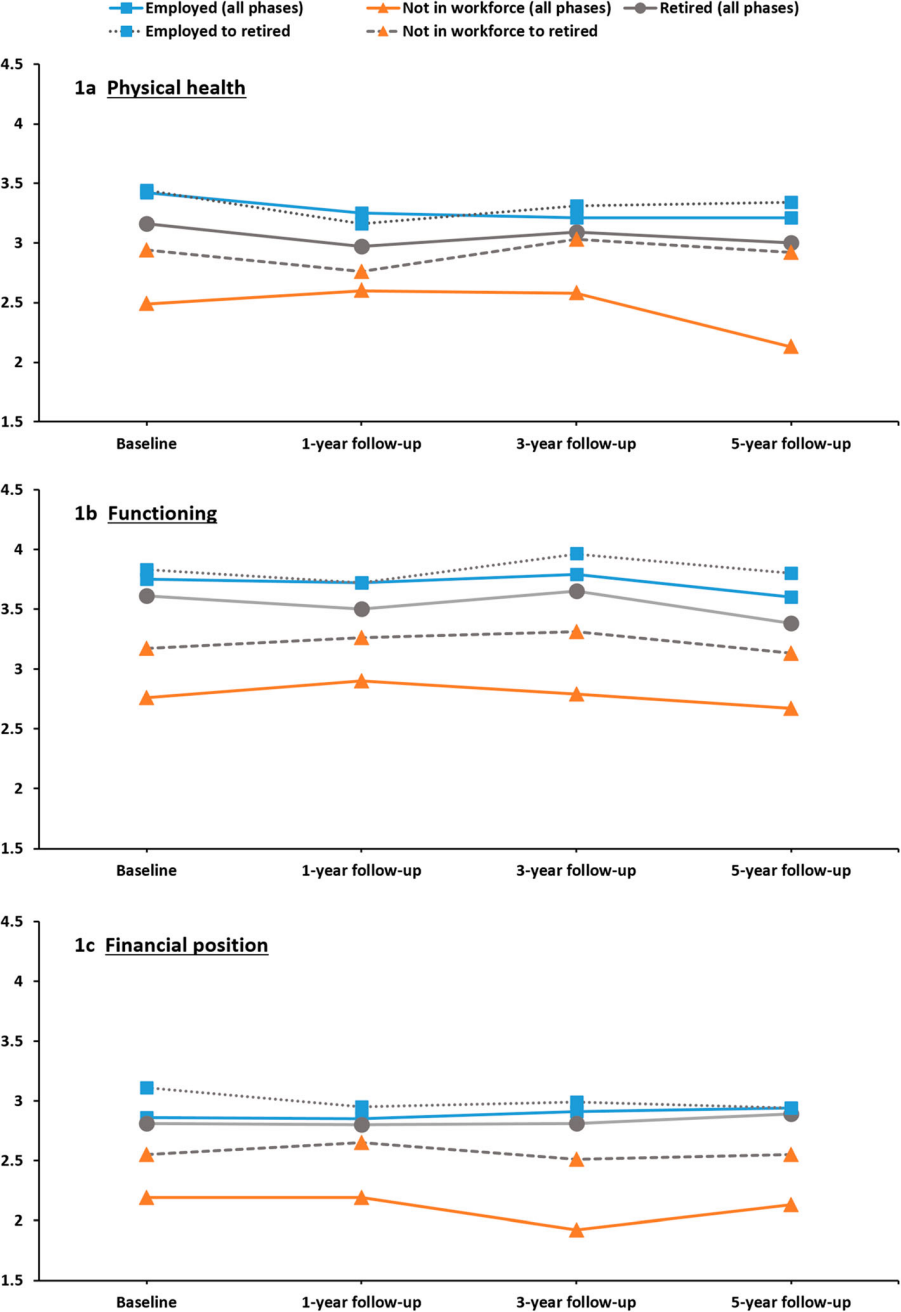
Outcome profiles by aggregate work/retirement status (*N* = 1931): **a** Physical health; **b** Functioning; and **c** Financial position. See Supplementary Table S2 for means (SDs)

It should also be noted that our primary interest was in assessing the significance or otherwise of individual predictors (and examining their profiles across the chosen global outcome domains), and not in the identification or testing of potential aggregate prediction equations or the modelling of inter-relationships between the outcomes; with the latter being a more appropriate course of action if we had comprehensive, independent measures of those outcomes, as opposed to a set of global self-reported perceptions. On the other hand, as most of the outcome measures were rated on comparable 1 to 5 scales, the raw score regression weights reported here are relatively easy to interpret and compare. So, by way of illustration (using the first regression weight of − 0.76 in Table 2): when controlling for the other predictor variables, perceived physical health was typically rated 0.76 units lower (on the global rating scale) by participants who were not in the workforce compared with those who were employed at all phases; this is comparable to the unadjusted mean difference between these groups across all phases of 0.80 (i.e., with corresponding group mean ratings of 2.50 and 3.30; see Supplementary Table S2 and Fig. 1a).

**Table 2:** Predictors of self-rated physical health, functioning and perceived financial position (*N* = 1903)

Predictor	Outcome variable – Global ratings (1 to 5):		
	Physical health	Functioning	Financial position
**Work/retirement status**			
Employed (all phases)	0	0	0
Not in workforce (all phases)	−0.76 (−0.99, −0.53)**	−0.89 (−1.14, −0.64)**	−0.55 (−0.75, −0.35)**
Retired (all phases)	−0.13 (−0.26, 0.00)	−0.08 (−0.21, 0.06)	−0.01 (−0.12, 0.09)
Employed to retired	0.07 (− 0.15, 0.28)	0.09 (− 0.15, 0.33)	0.24 (0.05, 0.43)*
Not in workforce to retired	− 0.36 (− 0.71, − 0.01)*	−0.50 (− 0.87, − 0.14)**	−0.26 (− 0.49, − 0.03)*
**Study phase**			
Baseline	0	0	0
1-year follow-up	−0.21 (− 0.31, − 0.10)**	−0.09 (− 0.19, 0.02)	−0.05 (− 0.12, 0.02)
3-year follow-up	− 0.21 (− 0.33, − 0.08)**	0.02 (− 0.11, 0.15)	0.03 (− 0.06, 0.12)
5-year follow-up	− 0.18 (− 0.31, − 0.06)**	− 0.15 (− 0.28, − 0.02)*	0.07 (− 0.03, 0.17)
**Significant work/retirement status by phase interactions**			
Employed to retired by 3-year follow-up			−0.18 (− 0.35, − 0.01)*
Employed to retired by 5-year follow-up			− 0.27 (− 0.47, − 0.08)**
**Sex**			
Male	0	0	0
Female	0.07 (− 0.04, 0.17)	0.08 (− 0.03, 0.19)	− 0.01 (− 0.10, 0.08)
**Live on a farm**			
No	0	0	0
Yes	0.03 (−0.09, 0.15)	−0.06 (− 0.18, 0.06)	−0.10 (− 0.20, 0.01)
**Any chronic disease**			
No	0	0	0
Yes	−0.33 (− 0.43, − 0.24)**	− 0.23 (− 0.33, − 0.13)**	−0.07 (− 0.15, 0.01)
**Currently married**			
No	0	0	0
Yes	0.11 (−0.01, 0.22)	0.20 (0.08, 0.32)**	0.28 (0.19, 0.38)**
**Recent adverse life events**	−0.13 (− 0.16, − 0.09)**	−0.15 (− 0.19, − 0.12)**	−0.07 (− 0.10, − 0.04)**
**ARIA**	−0.02 (− 0.03, − 0.01)*	−0.02 (− 0.03, 0.00)	− 0.02 (− 0.03, − 0.01)*
**Occupation**			
Manager	0	0	0
Professional	0.09 (−0.07, 0.26)	0.02 (− 0.15, 0.19)	0.21 (0.07, 0.36)**
Technician/trade worker	−0.09 (− 0.28, 0.10)	−0.12 (− 0.32, 0.08)	−0.16 (− 0.34, 0.03)
Community or personal service worker	− 0.07 (− 0.29, 0.14)	0.02 (− 0.21, 0.24)	− 0.17 (− 0.35, 0.01)
Clerical or administrative worker	− 0.08 (− 0.25, 0.10)	− 0.09 (− 0.28, 0.09)	−0.05 (− 0.21, 0.10)
Sales worker	− 0.03 (− 0.30, 0.24)	0.03 (− 0.23, 0.29)	− 0.07 (− 0.27, 0.14)
Machinery operator or driver	− 0.19 (− 0.43, 0.05)	−0.14 (− 0.43, 0.14)	−0.30 (− 0.53, − 0.07)*
Labourer	−0.18 (− 0.40, 0.05)	−0.16 (− 0.39, 0.07)	−0.21 (− 0.40, − 0.03)*
Not in workforce	−0.19 (− 0.41, 0.03)	−0.09 (− 0.31, 0.14)	−0.07 (− 0.28, 0.15)
Mixed occupations	− 0.08 (− 0.28, 0.13)	0.01 (− 0.20, 0.22)	−0.18 (− 0.35, − 0.01)*
Missing	−0.11 (− 0.35, 0.14)	−0.09 (− 0.36, 0.17)	−0.18 (− 0.38, 0.01)

*Note*: ARIA, Accessibility/Remoteness Index of Australia Plus. Tabled values are raw regression weights (99% CIs) from Generalised Estimating Equations, which controlled for participant ID and household ID (*N* = 1903 individuals; 1427 households; 4914 cases): *p < 0.01; **p < 0.001. Twelve predictors examining work/retirement status by phase interactions were also included in each analysis. A small number of individuals (*N* = 28) were excluded from these analyses due to missing scores on some of the predictor variables

A cross-sectional sub-analysis of self-reported reasons for retirement was also conducted based on respondents to the 5-year follow-up survey who were already retired, or planned to retire within the next 5 years. As detailed below, the aggregate work/retirement status groups examined in the main analyses, and the timeframe categories used in the self-reported reasons for retirement analyses, effectively had an age component built into them; consequently, age was not included as a predictor (or covariate) in any of these analyses. Significance was set at *p* < 0.01 as a partial control for the number of statistical tests.

## Results

### Participant characteristics

At baseline, 2013 participants met inclusion criteria for the current analyses (i.e., were aged 45 or over and had completed questions regarding their employment/retirement status). Over the four survey phases, there were a total of 5379 responses, with participants completing an average of 2.8 surveys in total. There were small but statistically significant correlations (ranging from 0.10 to 0.14, *p* < 0.001) between baseline ratings for five of the outcomes (except perceived relationships) and the number of survey phases completed; collectively, they accounted for 3.1% of the variance in this study retention index, with participants who perceived their baseline health and wellbeing more positively likely to complete more phases. By comparison, baseline outcomes ratings were moderately correlated (*p* < 0.001) with the corresponding ratings at subsequent phases (ranging from 0.52 to 0.58 for the global health ratings, and 0.69 for perceived financial position).

Participant characteristics are shown in Table 1 by data source (since different subsets of data were used in the various analyses). The majority (78.4%) of the sample were either employed or retired across all available phases, with a further 7.4% out of the workforce across all phases (e.g., unemployed, unable to work). Only two of the remaining aggregate work/retirement status groups had sufficient sample sizes at baseline to be retained in the primary analyses, including 6.8% of participants (*N* = 136) transitioning from employment to retirement across the study phases, and 3.4% (*N* = 68) who transitioned from not in the workforce to retirement. Consequently, only 10.6% of participants included in the primary analyses (*N* = 204 of 1931) actually transitioned to retirement during the study; amongst whom, 4.9, 58.3 and 36.8% were identified as transitioning by the 1-, 3-, and 5-year follow-ups, respectively.

There was a clear age progression across the retained groups, reflective of their delineation. On average (using all available data), participants who were employed (mean age = 55.54, SD = 6.66) or not in the workforce (mean age = 55.22, SD = 5.36) at all phases were younger, followed by the two transitioning groups (employed to retired: mean age = 62.65, SD = 5.31; not in workforce to retired: mean age = 61.14, SD = 3.85), with those retired at all phases being much older (mean age = 71.99, SD = 6.85). Other socio-demographic differences between the retained groups were in the expected directions (see Supplementary Table S1); for example, participants who were employed at all phases were more likely to live on a farm (32.6% vs. 12.9%) and reported lower aggregate rates of chronic disease (43.1% vs. 69.5%), largely reflective of our rural sampling and age-related effects. As shown in Table 1, the average age of the sample also rose by 4.16 years from baseline to the 5-year follow-up, while rates of chronic disease increased from half (53.1%) to two-thirds (66.5%) of the sample during this period, with blood pressure problems being the largest contributor (increasing from 40.0% at baseline to 53.4% at 5-years).

**Fig. 2:**
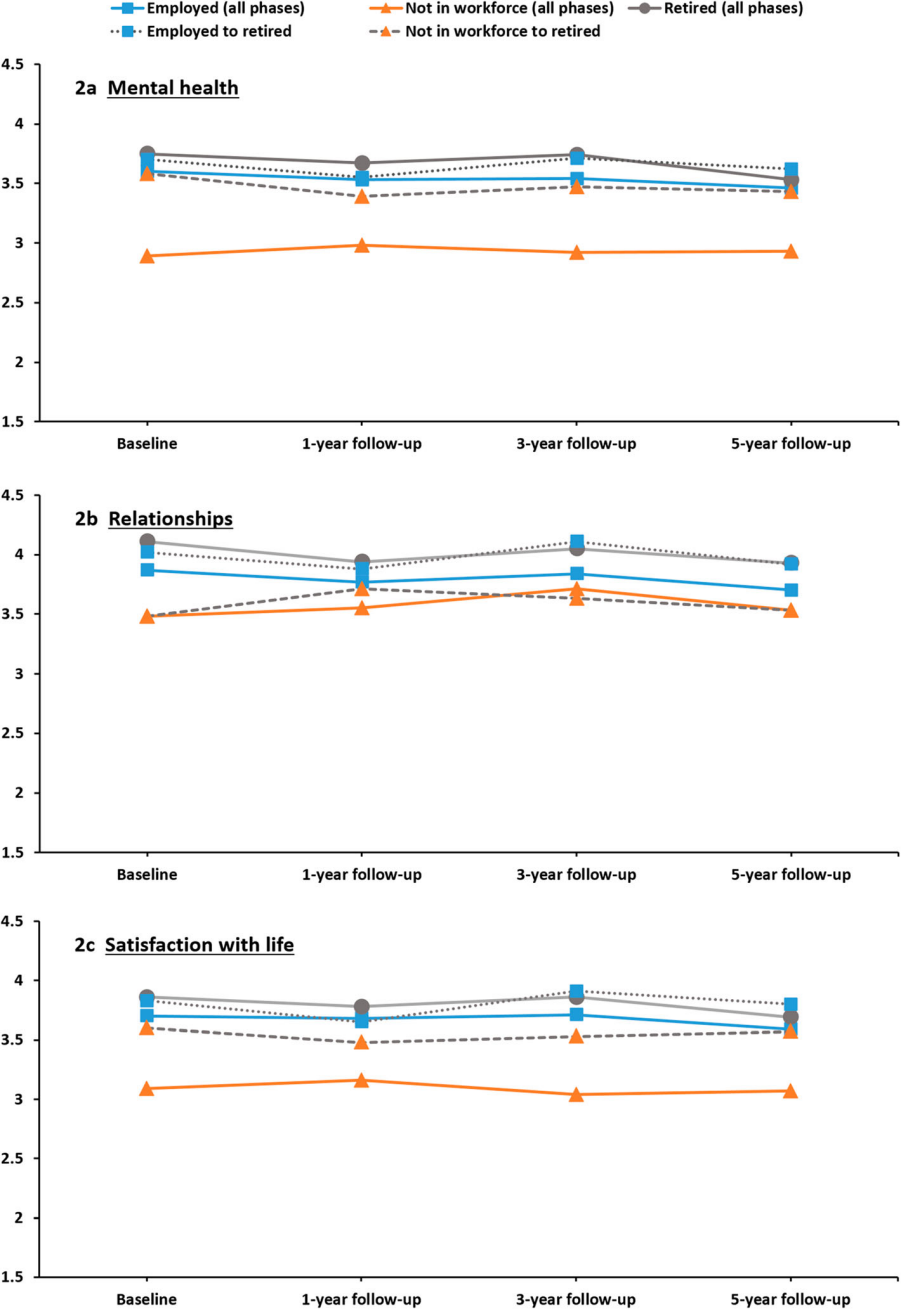
Outcome profiles by aggregate work/retirement status (*N* = 1931): **a** Mental health; **b** Relationships; and **c** Satisfaction with life. See Supplementary Table S2 for means (SDs)

Based on grand means for the global health ratings, ARMHS participants (aged 45 or over) viewed their ‘satisfaction with relationships’ most positively (3.93, SD 0.91), followed by three comparable outcome domains (satisfaction with life: 3.72, SD 0.88; mental health: 3.61, SD 1.00; and everyday functioning: 3.61, SD 1.04), with physical health (3.23, SD 0.99) receiving the lowest ratings. Supplementary Table S2 presents outcome variable profiles (means, SDs) across study phases for the five aggregate work/retirement status categories retained in the major GEE analyses; these profiles are also illustrated in Fig. 1 (physical/functional outcomes) and Fig. 2 (social/emotional outcomes).

### Physical/functional outcomes: physical health, everyday functioning, and financial position

Table 2 summarises the GEE analyses for the prediction of physical health, everyday functioning, and financial position. There was no significant difference between participants who were employed at all study phases and those retired at all study phases, or between those who were employed at all study phases and those who transitioned from employment to retirement during the study, with one exception. Participants who transitioned from employment to retirement during the study rated significantly higher on their overall perceived financial wellbeing than participants who remained in the workforce for the entire study period (with means of 3.00 vs. 2.88). However, there were also significant interaction effects between employment/retirement status and study phase for financial position (see Table 2), with a relative decline in perceived financial position over time among participants who moved from being employed to retired across the course of the study (see Table S2 and Fig. 1).

Those who were not in the workforce at all study phases, or transitioned from not in the workforce to retired, rated significantly lower on each of the three physical/functional outcomes (see Table 2) compared to employed participants (e.g., corresponding Table S2 grand means for everyday functioning: 2.79 vs. 3.22 vs. 3.73; see Fig. 1b). However, these effects tended to be less marked for those who transitioned from not in the workforce; corresponding regression weights (and 99% CIs) with not in the workforce as the reference category: physical health, 0.40 (0.00, 0.81), *p* = 0.010; everyday functioning, 0.38 (− 0.04, 0.81), *p* = 0.019; and financial position, 0.29 (0.00, 0.57), *p* = 0.009. This suggests that the transition to retirement (and/or anticipated transition) for those not in the workforce may have had some positive or stabilising effects for these outcomes relative to those who were not in the workforce at all phases. Alternatively, other characteristics of the latter group (not examined in the current analyses) may have contributed to their poorer baseline physical/functional wellbeing (see Table S2), which may have kept them out of the workforce for an extended period.

Among the other factors that were controlled for in the GEE analyses, several significant effects were observed, which were largely congruent with expectations based broadly on the psychosocial determinants research literature. A greater number of recent adverse events was associated with poorer outcomes for all three measures, while chronic disease was associated with poorer physical health and everyday functioning. Being currently married was associated with improved everyday functioning and financial position, while greater remoteness was associated with poorer physical health and financial position. Occupation was also significantly associated with financial position, with professionals scoring higher on this outcome than managers (the reference category), and machinery operators, labourers, and those reporting mixed occupations scoring lower. Physical health also tended to be rated higher at baseline, which is consistent with the changing rates of chronic disease noted earlier. Similarly, overall functioning was rated marginally lower at the 5-year follow-up.

### Social/emotional outcomes: mental health, relationships, and satisfaction with life

As detailed in Table 3, for the outcomes of mental health, satisfaction with relationships, and satisfaction with life, retired participants rated significantly more positively than those who were in the workforce, while those who were not in the workforce at all phases had significantly lower scores than employed participants on each of these measures (e.g., corresponding Table S2 grand means for mental health: 3.69 vs. 3.55 vs. 2.92; see Fig. 2a). There were no significant differences between employed participants and those who transitioned to retirement (either from employment, or from not being in the workforce). However, in accord with the earlier analyses, those who transitioned from not in the workforce to retired self-rated their mental health and satisfaction with life more positively than those not in the workforce at all phases; corresponding regression weights (and 99% CIs): mental health, 0.62 (0.21, 1.03), *p* < 0.001; satisfaction with life, 0.44 (0.08, 0.81), *p* = 0.002.

**Table 3:** Predictors of self-rated mental health, relationships and satisfaction with life (*N* = 1903)

Predictor	Outcome variable – Global ratings (1 to 5):		
	Mental health	Relationships	Satisfaction with life
**Work/retirement status**			
Employed (all phases)	0	0	0
Not in workforce (all phases)	−0.55 (− 0.82, − 0.28)**	−0.28 (− 0.53, − 0.04)*	−0.50 (− 0.73, − 0.26)**
Retired (all phases)	0.19 (0.06, 0.32)**	0.22 (0.10, 0.34)**	0.16 (0.05, 0.27)**
Employed to retired	0.11 (−0.12, 0.34)	0.14 (− 0.06, 0.34)	0.13 (− 0.05, 0.32)
Not in workforce to retired	0.07 (−0.27, 0.40)	− 0.36 (− 0.73, 0.01)	−0.05 (− 0.36, 0.25)
**Study phase**			
Baseline	0	0	0
1-year follow-up	−0.14 (− 0.24, − 0.03)*	− 0.18 (− 0.27, − 0.09)**	− 0.09 (− 0.17, 0.01)
3-year follow-up	− 0.09 (− 0.21, 0.04)	− 0.08 (− 0.19, 0.04)	− 0.03 (− 0.13, 0.08)
5-year follow-up	− 0.15 (− 0.28, − 0.03)*	− 0.22 (− 0.34, − 0.10)**	−0.12 (− 0.24, − 0.01)*
**Significant work/retirement status by phase interactions**			
Not in workforce to retired by 1-year follow-up		0.35 (0.04, 0.66)*	
**Sex**			
Male	0	0	0
Female	0.00 (− 0.11, 0.11)	0.10 (0.01, 0.20)*	0.06 (−0.04, 0.15)
**Live on a farm**			
No	0	0	0
Yes	0.02 (−0.11, 0.15)	−0.02 (− 0.13, 0.10)	−0.05 (− 0.15, 0.06)
**Any chronic disease**			
No	0	0	0
Yes	−0.12 (−0.23, − 0.02)*	−0.03 (− 0.12, 0.06)	−0.05 (− 0.13, 0.04)
**Currently married**			
No	0	0	0
Yes	0.18 (0.06, 0.30)**	0.15 (0.04, 0.26)**	0.23 (0.13, 0.34)**
**Recent adverse life events**	−0.20 (− 0.23, − 0.16)**	−0.16 (− 0.20, − 0.13)**	− 0.18 (− 0.21, − 0.14)**
**ARIA**	−0.01 (− 0.03, 0.00)	0.01 (− 0.01, 0.02)	0.00 (− 0.02, 0.01)
**Occupation**			
Manager	0	0	0
Professional	−0.01 (− 0.19, 0.17)	− 0.02 (− 0.18, 0.13)	−0.02 (− 0.18, 0.13)
Technician/trade worker	− 0.14 (− 0.35, 0.07)	− 0.16 (− 0.36, 0.03)	−0.11 (− 0.29, 0.07)
Community or personal service worker	− 0.09 (− 0.31, 0.13)	−0.02 (− 0.22, 0.18)	−0.01 (− 0.19, 0.18)
Clerical or administrative worker	− 0.05 (− 0.24, 0.14)	−0.06 (− 0.23, 0.11)	−0.09 (− 0.24, 0.07)
Sales worker	− 0.05 (− 0.34, 0.25)	0.01 (− 0.24, 0.27)	0.00 (− 0.24, 0.24)
Machinery operator or driver	− 0.18 (− 0.47, 0.11)	− 0.16 (− 0.44, 0.12)	−0.07 (− 0.31, 0.18)
Labourer	− 0.15 (− 0.37, 0.07)	− 0.16 (− 0.37, 0.05)	−0.13 (− 0.32, 0.07)
Not in workforce	− 0.05 (− 0.28, 0.19)	− 0.07 (− 0.27, 0.14)	−0.06 (− 0.25, 0.13)
Mixed occupations	− 0.03 (− 0.26, 0.19)	0.05 (− 0.14, 0.25)	− 0.07 (− 0.26, 0.13)
Missing	− 0.20 (− 0.44, 0.04)	−0.11 (− 0.34, 0.12)	−0.09 (− 0.27, 0.10)

*Note*: ARIA, Accessibility/Remoteness Index of Australia Plus. Tabled values are raw regression weights (99% CIs) from Generalised Estimating Equations, which controlled for participant ID and household ID (*N* = 1903 individuals; 1427 households; 4914 cases): *p < 0.01; **p < 0.001. Twelve predictors examining work/retirement status by phase interactions were also included in each analysis. A small number of individuals (*N* = 28) were excluded from these analyses due to missing scores on some of the predictor variables

There was also a significant interaction between employment/retirement status and study phase for the relationships outcome; perceived relationships tended to improve somewhat at 12 months for those who transitioned from not being in the workforce to being retired (from 3.48 to 3.55), relative to those who were in the workforce during all phases (from 3.87 to 3.77, see Table S2 and Fig. 2b). The relative benefits for retired participants were similar across the three outcomes. For participants who were not in the workforce, comparable deficits were found (relative to the reference group) for mental health (regression weight, − 0.55) and satisfaction with life (regression weight, − 0.50), with a weaker effect shown for relationships (regression weight, − 0.28). Indeed, within those who were not in the workforce for all phases, perceived relationships clearly received the highest mean ratings (3.52) relative to the other outcomes (2.16 to 3.10).

Other factors that were controlled for in the GEE analyses also had significant relationships with the social/emotional outcomes. Being currently married and having fewer adverse life events were both associated with higher scores on all three outcomes, while females scored slightly higher on their relationships, and those with any chronic disease reported poorer mental health. There was also a modest tendency for mental health and relationships to receive higher ratings at baseline, but this effect was inconsistent, with minimal differences at the 3-year follow-up. Satisfaction with life was also rated marginally lower at the 5-year follow-up, which was consistent with the other social/emotional outcomes.

### Self-reported reasons for retirement

As detailed in Table 4, the sub-analysis of self-reported reasons for retirement comprised 557 respondents to the 5-year follow-up survey, categorised by their retirement timeframe. Participants who retired more than 5 years before the study tended to report a younger retirement age, particularly female respondents (53.29 vs. 58.95 years), which is consistent with national retirement age trends. Overall, the most frequently given reason (35.9%) for retiring or being likely to retire was reaching the usual or desired retirement age. There were some notable differences between the reasons for retirement reported by those who had already retired (either prior to the study, or during the study phase) and the proposed reasons for retirement among those who planned to retire in the next 5 years. Eligibility for superannuation (or being financially secure) was listed as a proposed reason for retirement by almost half (44.2%) of those who planned to retire in the next 5 years, but was listed as an actual reason for retirement by only 19.1% of participants who were already retired. Similarly, lifestyle reasons and a declining interest in work were listed as proposed reasons for retirement by one-third (33.7%) and one-quarter (25.6%) of participants who were yet to retire, respectively, with significantly fewer retired participants indicating that these factors actually influenced their retirement decision (17.6 and 11.3%, respectively); these two reasons also showed the clearest progression across the four retirement timeframes in Table 4. Participants who had actually retired were also significantly more likely to report that this was due to their own sickness, injury, or disability than those who were yet to retire (15.3% vs. 3.5%).

**Table 4:** Reasons for retiring by retirement timeframe: subset of respondents to the 5-year follow-up survey (*N* = 557)

	Retired before baseline:		C. Retired during study period (*N* = 185)	D. Plan to retire in next 5 years (*N* = 86)	***p***
	A. > 5 years (*N* = 193)	B. Up to 5 years (*N* = 93)			
	% (n)	% (n)	% (n)	% (n)	
**Age** Mean (SD)	75.81 (6.24)	69.76 (4.67)	64.97 (4.59)	60.85 (4.80)	
**Sex**					
Male	42.0 (81)	49.5 (46)	42.7 (79)	30.2 (26)	0.071
Female	58.0 (112)	50.5 (47)	57.3 (106)	69.8 (60)	
Reached usual (or desired) retirement age	35.2 (68)	40.9 (38)	28.6 (53)	47.7 (41)	0.015
Eligibility for Old Age or other pension	12.4 (24)	18.3 (17)	11.4 (21)	15.1 (13)	0.402
Eligibility for superannuation (or financially secure)	17.1 (33)	18.3 (17)	21.6 (40)	44.2 (38)	< 0.001**
Own sickness, injury or disability	14.0 (27)	8.6 (8)	20.0 (37)	3.5 (3)	0.001*
Stress or mental health problem	5.7 (11)	2.2 (2)	9.7 (18)	5.8 (5)	0.095
Own business closed or sold	8.8 (17)	21.5 (20)	13.0 (24)	10.5 (9)	0.022
No paid work available	2.1 (4)	3.2 (3)	3.8 (7)	0.0 (0)	0.286
Made redundant/dismissed	9.8 (19)	4.3 (4)	5.9 (11)	1.2 (1)	0.034
Declining interest in work	5.2 (10)	15.1 (14)	15.7 (29)	25.6 (22)	< 0.001**
Retirement of partner	15.0 (29)	8.6 (8)	9.2 (17)	23.3 (20)	0.006*
Lifestyle reasons	14.0 (27)	18.3 (17)	21.1 (39)	33.7 (29)	0.002*
To care for family member/friend	9.8 (19)	3.2 (3)	7.0 (13)	2.3 (2)	0.056
To undertake voluntary work	2.1 (4)	5.4 (5)	8.6 (16)	7.0 (6)	0.043

*Note*: In Australia, ‘superannuation’ typically refers to money compulsorily put aside by your employer over your working life that you can access when you retire (currently equivalent to a minimum of 9.5% of your salary). The four categories reported here were based on employment status at baseline, any subsequent retirement during the study period, and self-reported duration and reasons for retirement from the 5-year follow-up survey (with a minimum of one reason required for inclusion in this analysis). Statistical significance was based on overall Chi-square tests (df = 3): *p < 0.01, **p < 0.001

In addition, we compared self-reported reasons for retirement provided by male and female respondents. Overall, males were more likely than females to list eligibility for superannuation (or being financially secure) as a reason for retirement (29.3% vs. 18.5%; χ^2^_(1)_ = 9.00, *p* = 0.003); however, this disparity was less marked among those who planned to retire in the next 5 years (46.2% vs. 43.3%). While males were also more likely to list their own sickness, injury, or disability as a reason for retirement (18.1% vs. 10.2%; χ^2^_(1)_ = 7.34, *p* = 0.007), this disparity was greatest among those who retired more than 5 years before the study (24.7% vs. 6.3%). The progression across categories noted in Table 4 for a declining interest in work was more evident among female respondents (2.7, 8.5, 15.1, 25.0%), although there was no overall difference in the endorsement of this reason between male and female respondents (15.9% vs. 11.7%; χ^2^_(1)_ = 2.10, *p* = 0.147). In contrast, two reasons for retirement were clearly less likely to be listed by male respondents, retirement of partner (1.7% vs. 21.5%; χ^2^_(1)_ = 46.13, *p* < 0.001) and caring for family member/friend (2.6% vs. 9.5%; χ^2^_(1)_ = 10.55, *p* = 0.001). Moreover, there were only 4 (of 232) male respondents who identified retirement of partner as one of their main reasons for retirement, 3 of whom were in the yet to retire subgroup.

Among participants included in the reasons for retirement analyses, there were 64 respondents (11.5%) who reported employment as a farmer in at least one survey. They differed significantly from non-farmers on only one of the reasons for retirement, with ‘own business closed or sold’ listed as a retirement reason by a quarter (25.0%) of the farmers compared with 11.0% of the non-farmers (χ^2^_(1)_ = 10.17, *p* = 0.001); however, this disparity was smaller among those who retired more than 5 years before the study (13.3% vs. 8.4%).

## Discussion

The aim of this paper was to explore the health and wellbeing of rural and remote Australians across various employment and retirement categories, whilst accounting for other psychosocial determinants. Across all six self-reported outcomes, respondents who were not in the workforce rated significantly more poorly than those who were employed, with mean scores also indicating that they rated more poorly than retired participants on each outcome. Recent longitudinal studies using large representative samples have similarly demonstrated that employed individuals have better self-rated health than unemployed people [38] and that most people sustain their pre-retirement self-rated health levels during the initial post-retirement years [39].

The important role of enduring and stable social supports (e.g., currently married; absence of associated relationship stressors) was reinforced in the current analyses, with marital status and recent adverse life events being significantly associated with most of the outcome measures, which is consistent with previous ARMHS findings [10, 14, 29]. The observed associations between remoteness and poorer physical health and financial position are also consistent with earlier findings of greater social disadvantage in remote communities [33].

Viewing the current data set as a whole, there was limited evidence for differential change from baseline in health and wellbeing perceptions across the work/retirement status groups (e.g., minimal interaction effects, indicating: a relative decline in perceived financial position among participants moving from employed to retired; and an initial improvement in perceived relationships among participants transitioning from not in the workforce to retired). Therefore, to a large extent, the window on work/retirement effects provided by these data is a relatively stable one, a multi-phase cross-section if you like, that probably reveals as much about baseline characteristics, long-term employment benefits, and associated capacity to retire effects as it does about recent or pending transitions. In short, the factors that contributed to non-participation in the workforce and/or to retirement decisions are likely to have had an ongoing impact on perceived health and wellbeing. However, these impacts may be less marked if workforce non-participation begins close to the usual or desired retirement age.

The above caveats notwithstanding, our results suggest that the context of retirement is important, and its effects on health and wellbeing differ depending on pre-retirement circumstances. For example, while participants who were not in the workforce for the duration of the study rated significantly poorer mental health, relationships, and satisfaction with life compared with employed participants, these differences tended to dissipate somewhat when participants transitioned from being not in the workforce to retirement. In Australia, the age pension has reasonably similar financial benefits to other government assistance schemes, such as disability benefits; although unemployment benefits are currently substantially lower (however, they were temporarily boosted during the first year of the COVID-19 pandemic). These findings are therefore less likely to be related to financial circumstances, and may reflect the importance of self-perception, sense of purpose, or stigma, and the effects that these may have on an individual’s wellbeing. Negative perceptions associated with receipt of unemployment benefits have also been shown to dissipate with re-entry to the workforce [40]; perhaps, similar reductions in self-perceived stigma occur with transition to an age pension.

Given the broad range of factors impacting on retirement decisions, it is not surprising that there is now considerable heterogeneity in retirement timing [41]. Previous research has also indicated that contextual differences play a role in individuals’ experience of retirement, resulting in inconsistent research findings regarding the effects of retirement on health and wellbeing. In NSW’s 45 and Up study, significant decreases were found in physical functioning for men and women following retirement, with men also reporting a small increase in their psychological distress during this time [42]. An earlier analysis of this data set showed that retirement had a negative effect on psychological distress for those aged 45–64, as well as for men aged 65–74, but that this effect did not occur in those aged 75 and older [43]. The authors, and others [16], speculated that such patterns may be attributable to involuntary retirement in younger age groups (e.g., due to physical health issues, redundancy, or caring responsibilities) and, therefore, may not be the result of retirement directly. Conversely, findings from both the 45 and Up and the Household, Income and Labour Dynamics in Australia (HILDA) studies have shown that retirement has a positive effect on health behaviours, including physical activity, smoking, and sleep [44, 45]. This improvement in health behaviours following retirement may negate some of the physical effects of aging, which may explain the current study’s findings that retirement had no effect on perceived physical health. Overall, findings from the HILDA survey suggest that there is a high level of diversity in people’s post-retirement experiences, with 60% of people experiencing fluctuations in life satisfaction following retirement [46]. In concordance with this, research from the Australian National Survey of Mental Health and Wellbeing concluded that there appears to be no categorical benefit or harm associated with retirement, in itself [47].

Several differences were observed between employed and retired participants across the phases in the current study. Notably, participants who transitioned from employment to retirement during the study initially reported a significantly higher perceived financial position than participants who remained in employment. This may reflect lifestyle changes in retirement that are associated with lower expenses, or may be a contributing factor in the decision to retire (i.e., people who feel more prosperous financially may believe they are in a better position to retire). However, there was a relative decrease in this effect over time, with the two groups reporting similar perceived financial positions by the conclusion of the study.

Retired participants also rated significantly higher mental health, satisfaction with relationships, and satisfaction with life, compare with participants who remained in the workforce across the study. However, caution needs to be exercised in drawing any causal inferences from the study’s findings, with the possibility of bi-direction associations for many of the factors assessed, or even reverse causality. For example, experiencing positive social/emotional wellbeing, and associated lifestyle factors, could contribute both to the decision to retire from the workforce and/or be a potential benefit arising from having made that transition.

Pre-retirement circumstances may still play a part in countries with universal aged pension schemes. A recent New Zealand study examining living standards trajectories for adults aged 55 to 76 [48] demonstrated clear relationships with concurrent mental and physical health. Those with ‘good, stable’ living standards (> 85% of the population) in the period preceding pension eligibility reported relatively stable mental health with age but moderate declines in physical health. By comparison, among subgroups characterised initially by hardship, those with ‘increasing’ living standards reported improvements in mental health with age but no change in physical health, whereas those with ‘decreasing’ living standards reported declining mental and physical health with age. Consequently, the wellbeing of disadvantaged adults could be disproportionately affected by increases in pension eligibility age [48].

When exploring actual and expected reasons for retirement, several significant differences were observed between respondents who had already retired and those who expected to retire in the near future. Those who were yet to retire were more likely to indicate that retirement decisions would be based on financial security or eligibility for superannuation, or due to a declining interest in work or other lifestyle reasons. Interestingly, the proportion of people who had retired for these reasons increased among those recently retired (e.g., 21% of respondents who retired during the study period reported that it was due to lifestyle reasons, compared with 14% of those who had been retired for longer than 5 years). Therefore, while the results of this analysis may reflect perceptions of retirement differing among those who are yet to retire, it may also indicate a genuine change over time in the circumstances surrounding the decision to retire, with control over this decision increasing compared to previous generations. Conversely, ongoing environmental stressors and associated financial hardship within the farming sector may have contributed to higher rates of exit from the industry in more recent times [24, 32].

The observed differences in reasons for retirement between male and female respondents appear to reflect a mixture of generational and cultural factors, with females tending to cite external factors that were rarely listed by males (e.g., retirement of partner, caring for others). Previous research by Atalay et al. [49] reported that a wife’s retirement has a positive effect on her husband’s mental health, and that this effect gets stronger over time. Men whose wife has retired are more likely to engage in voluntary work, socialise more regularly, become an active member of a club or organisation, and are more likely to retire themselves. Consequently, while males and females may report different reasons for retirement, some of the benefits of retirement may still be shared.

On the other hand, eligibility for superannuation and declining interest in work were just as likely to be mentioned by male and female respondents who plan to retire in the next 5 years. This may reflect changing work patterns for females in recent years, with a higher proportion likely to be engaged in full-time work than in previous generations, making them more able to make financially based retirement plans. One other area of difference between males and females in the current study was the tendency for females to report better social relationships than their male counterparts. Previous research has shown that engaging in social groups following retirement increases life expectancy [50], while Olesen et al. [51] reported that there is a differential benefit of social relationships on mental health for retired compared with employed individuals, particularly for those who are younger when they retire.

This study has several limitations. The outcomes were single-item self-report measures and may not be as valid as longer, multiple-item measures. Retirement versus age-based or population cohort effects were unable to be clearly differentiated in the current study. Consequently, the positive retirement effects in the social/emotional outcomes analyses may reflect a mixture of retirement, ageing and/or generational impacts. In addition, only half (47.4%) of the baseline participants completed the five-year follow-up survey, and consequently selective attrition may have occurred, such that the perceived health and wellbeing profiles and reported reasons for retirement are unrepresentative. The reasons for retirement may have also been influenced by recall bias, particularly among those who were retired for more than 5 years.

Other multivariate analysis strategies may have also been worth considering, particularly if a higher proportion of the sample had experienced work/retirement transitions during the study phases and reasons for these decisions, and associated factors, had been prospectively assessed at each survey phase. Under such circumstances, analytical techniques like GLMM (Generalised Linear Mixed Models) could have been used to more fully explore within-subject profiles, such as ‘when and why’ particular transitions occurred, and the magnitude and timing of associated impacts on the outcomes of interest. However, in the current study, self-reported reasons for actual retirement, or planned future retirement, were only elicited during the 5-year follow-up survey.

Finally, with respect to potential policy implications arising from the current study, we offer two modest suggestions:

Firstly, being an active member of the workforce, physically healthy, and having stable social supports all contribute positively to self-reported health and wellbeing. However, while these factors are likely to be equally as important in urban and rural settings, the rates at which they co-occur are likely to be different, as are the systems, strategies and resources needed to counter their absence. We need to continue to advance policies and programs that address the higher rates of unemployment and underemployment, job insecurity, income inequality, and disadvantage experienced in rural and remote areas [52], together with the higher rates of health problems and reduced access to health services [53, 54]. Arguably, localised strategies also need to be fostered within rural communities that address social connection, mental health problems, early intervention, service accessibility, and health workforce challenges [54]. More broadly, policies that promote identification and alleviation of poor health and material hardship in the decade prior to retirement (or pension/superannuation eligibility) may reduce health inequalities in later life [48]. Such imperatives are likely to be even more important in this post-COVID-19 world, including ongoing job guarantees and universal basic income schemes [55].

Secondly, our examination of actual and expected reasons for retirement suggests that those who were yet to retire tended to have relatively narrow and overly optimistic expectations about this transition (e.g., relating to financial security, and changing work and lifestyle interests). Given the broad range of personal, contextual and societal factors that can impact on workforce retention and retirement planning, we probably need to redirect our public education programs away from their historical focus on age and financial security considerations in retirement planning and place greater emphasis on individual experiences, diversity and flexibility (including valuing non-paid community roles such as caring and mentoring). Previous World Health Organization campaigns emphasising ‘active ageing’, and more recent ones promoting the *Decade of Healthy Ageing* (2020–2030), provide useful positive frameworks for promoting such diversity, sharing expertise, and reducing ageism [56]. Providing encouragement and opportunities for older workers to be retained in the Australian workforce will also require a range of interconnected policy adjustments [57].

## Conclusions

Many of the contextual effects surrounding retirement have ongoing impacts, while others may affect an individual’s health and wellbeing in the short term only. In light of this, we need to arrive at a balance of supports for older people during this time of transition. Knowledge about the influence of contextual factors could assist governments in providing broader supports for older people who are not participating in the workforce prior to retirement age, as well as those entering retirement. One approach to establishing this support is to provide a variety of roles for older people, including volunteering, part-time and casual employment, and other community service activities, to enable them to maintain important social connections and sense of purpose during the initial retirement phase.

The findings from this study suggest that while retirement is a significant life transition that may affect multiple facets of an individual’s life, the direction and magnitude of these effects vary depending on the retirement context, namely the pre-retirement and concurrent circumstances within which an individual is retiring, including accessibility of services and supports. Personal perceptions of status changes may also contribute to an individual’s wellbeing, more so than objective factors such as income. Holistic policies and programs are needed that simultaneously promote rural work/retirement opportunities and diversity and address rural disadvantage.

## Supplementary Information

**Additional file 1: Supplementary Table S1.** Aggregate participant characteristics (across phases) by work/retirement status for the retained groups (*N* = 1931 individuals; 5072 surveys). **Supplementary Table S2.** Outcome variable profiles (Means, SD) by aggregate work/retirement status and phase (*N* = 1931).

## Data Availability

The datasets used and/or analysed during the current study are available from the ARMHS Chief Investigator (CI) on reasonable request (brian.kelly@newcastle.edu.au). The data are not publicly available due to ethical requirements, but may be accessed with necessary administrative ethical approval. All authors are ARMHS investigators and had direct access to the data.

## Abbreviations

ABS: Australian Bureau of Statistics
ARIA+: Accessibility/Remoteness Index of Australia Plus
ARMHS: Australian Rural Mental Health Study
ANZSCO: Australian and New Zealand Standard Classification of Occupations
GEE: Generalised estimating equation
NSW: New South Wales
