# Supplementary material for: Employment and retirement impacts on health and wellbeing among a sample of rural Australians
Source: BMC Public Health. 2021 May 10;21:888. doi: 10.1186/s12889-021-10876-9 (PMC8108016; doi:10.1186/s12889-021-10876-9)
Supplement: Supplementary file 1 — Additional file 1: Supplementary Table S1. Aggregate participant characteristics (across phases) by work/retirement status for the retained groups (N = 1931 individuals; 5072 surveys). Supplementary Table S2. Outcome variable profiles (Means, SD) by aggregate work/retirement status and phase (N = 1931). [file 12889_2021_10876_MOESM1_ESM.pdf]

Supplementary Table S1

Aggregate participant characteristics (across phases) by work/retirement status for the retained groups (N = 1,931 individuals; 5,072 surveys).

	**Work/retirement status**				
**Characteristic**	**Employed (all phases)** [2,085 surveys]	**Not in workforce (all phases)** [246 surveys]	**Retired (all phases)** [1,980 surveys]	**Employed to retired** [506 surveys]	**Not in workforce to retired** [255 surveys]
**Age** Mean (SD)	55.54 (6.66)	55.22 (5.36)	71.99 (6.85)**	62.65 (5.31)**	61.14 (3.85)**
**Sex**					
Male	44.7%	45.5%	39.8%*	42.1%	32.2%**
Female	55.3%	54.5%	60.2%	57.9%	67.8%
**Live on a farm**					
No	67.4%	85.1%**	89.4%**	77.8%**	89.6%**
Yes	32.6%	14.9%	10.6%	22.2%	10.4%
**Any chronic disease**					
No	56.9%	47.6%*	25.8%**	38.7%**	34.1%**
Yes	43.1%	52.4%	74.2%	61.3%	65.9%
**Currently married**					
No	17.7%	31.8%**	30.4%**	16.3%	31.8%**
Yes	82.3%	68.2%	69.6%	83.7%	68.2%
**Recent adverse life events** Mean (SD)	1.19 (1.20)	1.58 (1.51)**	0.98 (1.13)**	1.15 (1.24)	1.31 (1.25)
0-2	86.5%	75.3%**	91.1%**	87.5%	83.5%
3-5	13.0%	21.4%	8.3%	12.1%	16.1%
6-9	0.5%	3.3%	0.7%	0.4%	0.4%
**ARIA** Mean (SD)	4.30 (3.29)	4.15 (3.39)	3.76 (2.94)**	3.66 (2.92)**	3.82 (3.12)
Inner regional	36.1%	39.4%	45.0%**	41.9%	43.9%*
Outer regional	37.6%	38.6%	33.7%	37.4%	38.8%
Remote	19.3%	13.4%	17.5%	16.2%	14.1%
Very remote	7.0%	8.5%	3.7%	4.5%	3.1%

Note: ARIA, Accessibility/Remoteness Index of Australia Plus. Reported statistical tests are based on comparisons with the ‘Employed (all phases)’ group (*Reference category*), comprising either paired chi-square tests or planned comparisons from Analyses of Variance: *$p < 0.01$; **$p < 0.001$. For example, the ‘Retired (all phases)’ group differed from the reference group on all of the tabled characteristics, being older, more likely to be female or to have a chronic disease, and less likely to be currently married, to live on a farm, to have reported recent adverse events, or to live in more remote locations.

Supplementary Table S2
Outcome variable profiles (Means, SD) by aggregate work/retirement status and phase (N = 1,931).

**Outcome variable [Global ratings (1 to 5)] by Work/retirement status**	**Data source:**				
	**Baseline**	**1-year follow-up**	**3-year follow-up**	**5-year follow-up**	**All phases**
**Physical health**:					
Employed (all phases)	3.42 (0.94)	3.25 (0.90)	3.21 (0.90)	3.21 (0.90)	3.30 (0.92)
Not in workforce (all phases)	2.49 (0.97)	2.60 (1.02)	2.58 (0.97)	2.13 (1.13)	2.50 (0.99)
Retired (all phases)	3.16 (0.97)	2.97 (0.91)	3.09 (0.92)	3.00 (0.90)	3.07 (0.94)
Employed to retired	3.44 (0.91)	3.16 (0.91)	3.31 (0.91)	3.34 (0.99)	3.31 (0.93)
Not in workforce to retired	2.94 (1.12)	2.76 (0.97)	3.03 (0.93)	2.92 (1.00)	2.91 (1.01)
**Functioning**:					
Employed (all phases)	3.75 (0.93)	3.72 (0.89)	3.79 (0.91)	3.60 (0.94)	3.73 (0.92)
Not in workforce (all phases)	2.76 (1.07)	2.90 (1.10)	2.79 (1.06)	2.67 (1.11)	2.79 (1.07)
Retired (all phases)	3.61 (1.05)	3.50 (1.04)	3.65 (1.01)	3.38 (1.01)	3.55 (1.04)
Employed to retired	3.83 (1.04)	3.72 (0.95)	3.96 (0.86)	3.80 (0.94)	3.82 (0.95)
Not in workforce to retired	3.17 (1.13)	3.26 (1.09)	3.31 (0.92)	3.13 (1.08)	3.22 (1.05)
**Financial position**:					
Employed (all phases)	2.86 (0.72)	2.85 (0.68)	2.91 (0.76)	2.94 (0.76)	2.88 (0.72)
Not in workforce (all phases)	2.19 (0.73)	2.19 (0.73)	1.92 (0.65)	2.13 (1.06)	2.16 (0.75)
Retired (all phases)	2.81 (0.76)	2.80 (0.70)	2.81 (0.68)	2.89 (0.68)	2.82 (0.72)
Employed to retired	3.11 (0.84)	2.95 (0.69)	2.99 (0.74)	2.94 (0.76)	3.00 (0.76)
Not in workforce to retired	2.55 (0.75)	2.65 (0.77)	2.51 (0.77)	2.55 (0.72)	2.56 (0.75)
**Mental health**:					
Employed (all phases)	3.60 (0.97)	3.53 (0.91)	3.54 (0.92)	3.46 (0.96)	3.55 (0.95)
Not in workforce (all phases)	2.89 (1.15)	2.98 (1.14)	2.92 (1.44)	2.93 (1.10)	2.92 (1.17)
Retired (all phases)	3.75 (0.93)	3.67 (0.96)	3.74 (0.94)	3.53 (0.90)	3.69 (0.94)
Employed to retired	3.70 (0.99)	3.55 (1.05)	3.71 (1.01)	3.62 (0.99)	3.65 (1.01)
Not in workforce to retired	3.58 (1.05)	3.39 (1.12)	3.47 (1.04)	3.43 (1.05)	3.47 (1.06)
**Relationships**:					
Employed (all phases)	3.87 (0.89)	3.77 (0.82)	3.84 (0.85)	3.70 (0.91)	3.81 (0.87)
Not in workforce (all phases)	3.48 (1.09)	3.55 (1.13)	3.71 (1.23)	3.53 (1.25)	3.52 (1.12)
Retired (all phases)	4.11 (0.83)	3.94 (0.86)	4.05 (0.84)	3.93 (0.89)	4.03 (0.85)
Employed to retired	4.02 (0.87)	3.88 (0.80)	4.11 (0.83)	3.92 (0.92)	3.98 (0.86)
Not in workforce to retired	3.48 (1.19)	3.71 (0.97)	3.63 (0.92)	3.53 (0.89)	3.59 (1.00)
**Satisfaction with life**:					
Employed (all phases)	3.70 (0.86)	3.68 (0.77)	3.71 (0.80)	3.59 (0.84)	3.68 (0.82)
Not in workforce (all phases)	3.09 (1.05)	3.16 (0.92)	3.04 (1.16)	3.07 (1.10)	3.10 (1.03)
Retired (all phases)	3.86 (0.79)	3.78 (0.80)	3.86 (0.77)	3.69 (0.83)	3.81 (0.80)
Employed to retired	3.83 (0.81)	3.65 (0.88)	3.91 (0.71)	3.80 (0.81)	3.79 (0.81)
Not in workforce to retired	3.60 (1.01)	3.48 (0.88)	3.53 (0.85)	3.57 (0.87)	3.55 (0.90)

Note: These outcome profiles are illustrated in Figures 1 and 2. There were moderate correlations between the global health ratings, with the strongest associations being between mental health and satisfaction with life (0.64), relationships (0.62) and functioning (0.59), and between physical health and functioning (0.65). Correlations with perceived financial status were lower, ranging from 0.20 to 0.32.
